# The effectiveness of electroconvulsive therapy in substance use disorder at pharmacological treatment failure major depression

**DOI:** 10.1192/j.eurpsy.2023.1192

**Published:** 2023-07-19

**Authors:** P. Dannon

**Affiliations:** psychiarty, Hebrew university, jerusalem, Israel

## Abstract

**Introduction:**

Treatment resistant depression (TRD) is common with substance use disorder (SUD) and few studies demonstrated the effectiveness of medication-psychotherapy treatments in this population

**Objectives:**

To compare the effeictiveness of ECT in the treatment resistant depression patients vs TRD with SUD patients.

**Methods:**

14 TRD patients with 14 TRD-SUD patients compared in terms of ECT treatment response rates at baseline, thhree months and six months of the follow up periond. Pateints completed Hamilton anxiety, Hamilton depression-21 items, Barrat 11 impulsivity and visual analog scales each follow up visit.

**Results:**

Both groups completed ECT treatment between 2011-2018 with follow up of 12.3+4.1 months following the ECT procedure. Patients received average 11.7+2.6 bilateral ECT treatments per series. Both groups responded well to ECT treatment in terms of response rates and side effects however there were higher rates of relapse at intermediate to long term follow up period at TRD-SUD group.

**Conclusions:**

ECT seems to be an effective treatment for patiens of TRD-SUD. Moreover the response rates are equal to treatment reisstant depression cases without substance use disorder.

**Disclosure of Interest:**

None Declared

